# Image-Quality–Aware Multimodal Artificial Intelligence for Automated Structured OCT Report Generation in Glaucoma Evaluation

**DOI:** 10.1016/j.xops.2026.101254

**Published:** 2026-05-26

**Authors:** Jalil Jalili, Yashraj Gavhane, Evan Walker, Anna Heinke, Christopher Bowd, Akram Belghith, Massimo A. Fazio, Christopher A. Girkin, C. Gustavo De Moraes, Jeffrey M. Liebmann, Sally L. Baxter, Robert N. Weinreb, Linda M. Zangwill, Mark Christopher

**Affiliations:** 1 Division of Ophthalmology Informatics and Data Science, Viterbi Family Department of Ophthalmology, Shiley Eye Institute, University of California San Diego, La Jolla, California; 2 Hamilton Glaucoma Center, Viterbi Family Department of Ophthalmology, Shiley Eye Institute, University of California San Diego, La Jolla, California; 3 Department of Computer Science and Engineering, University of California San Diego, La Jolla, California; 4 Department of Ophthalmology and Vision Sciences, University of Alabama at Birmingham, Birmingham, Alabama; 5 Department of Ophthalmology, Harkness Eye Institute, Bernard and Shirlee Brown Glaucoma Research Laboratory, New York, New York

**Keywords:** Multimodal large language model, Glaucoma detection, Retinal nerve fiber layer, Quality triage, Clinical report generation

## Abstract

**Objective:**

To develop an explainable multimodal large language model (MM-LLM) that (1) screens optic nerve head (ONH) OCT circle scans for quality and (2) generates structured clinical reports that include glaucoma diagnosis and sector-wise retinal nerve fiber layer (RNFL) thinning assessments.

**Design:**

A retrospective cohort study using longitudinal data from the Diagnostic Innovations in Glaucoma Study and the African Descent and Glaucoma Evaluation Study.

**Participants:**

A total of 43 849 Spectralis circumpapillary B-scans centered on the ONH from 1310 subjects, including 1331 glaucomatous and 867 healthy eyes.

**Methods:**

An MM-LLM (Llama 3.2 Vision-Instruct model) was fine-tuned to generate clinical descriptions of OCT imaging data. Training data included paired OCT images and automatically generated, structured clinical reports that described global and sectoral RNFL thinning. Poor-quality scans were labeled as unusable and paired with a fixed refusal statement. The model was evaluated on a held-out test set for 3 tasks: quality assessment, glaucoma detection, and RNFL thinning classification across 7 anatomical sectors. Evaluation metrics included accuracy, sensitivity, specificity, precision, and F1-score. Model description quality was also evaluated using standard text evaluation metrics (BLEU, ROUGE, METEOR, and BERTScore).

**Main Outcome Measures:**

Diagnostic accuracy metrics for each task; text evaluation metrics for description quality.

**Results:**

The model achieved 0.90 accuracy and 0.98 specificity for quality triage. For glaucoma detection, accuracy was 0.86 (sensitivity 0.93, specificity 0.65, and F1-score 0.91). Retinal nerve fiber layer thinning prediction accuracy ranged from 0.83 to 0.94, with the highest performance in global, temporal, temporal superior, and temporal inferior sectors. Text generation scores (mean ± standard deviation) showed strong alignment with reference reports (BLEU: 0.82 ± 0.19; ROUGE-1: 0.94 ± 0.08; ROUGE-2: 0.87 ± 0.17; ROUGE-L: 0.92 ± 0.11; BERTScore-F1: 0.99 ± 0.02). Stratified analysis revealed better RNFL thinning detection in moderate-to-advanced glaucoma cases, especially in temporal sectors, while performance in nasal regions was better for mild cases.

**Conclusions:**

The fine-tuned MM-LLM generated accurate clinical descriptions based on OCT imaging. The model achieved high accuracy in identifying image quality issues and detecting glaucoma. The model provided sectoral descriptions of RNFL thinning to support clinical OCT evaluation. This approach shows potential as a scalable tool for clinical decision support, but further validation across additional datasets is needed.

**Financial Disclosure(s):**

Proprietary or commercial disclosure may be found in the Footnotes and Disclosures at the end of this article.

Glaucoma is progressive optic neuropathy and a leading cause of irreversible blindness worldwide.^1^ Early detection, particularly of retinal nerve fiber layer (RNFL) thinning, is critical for preserving vision, with OCT serving as a key imaging modality.^2^ OCT-derived RNFL measurements provide essential evidence of glaucomatous structural damage, often before functional loss appears in visual field (VF) testing.^3^ Although OCT enables early structural assessment, interpretation can be impeded by poor image quality and relies heavily on clinician expertise, especially when thinning patterns are subtle or complicated by comorbidities.^4^ In addition, electronic health record documentation is a long-recognized burden for physicians and a known contributor to physician burnout,^5^ with ophthalmologists facing particular challenges due to the high volume of patient visits and severe time constraints.^6^^,^^7^

To address these challenges, artificial intelligence (AI) models have been proposed to assist in glaucoma detection and OCT interpretation.^8^, ^9^, ^10^, ^11^, ^12^ While convolutional neural networks have demonstrated success in classification tasks, they offer limited explainability and interpretability and are generally restricted to binary or quantitative predictions.^13^^,^^14^ More recently, vision-language models and multimodal large language models (MM-LLMs) have emerged as promising tools for clinical applications, enabling the generation of free-text explanations based on imaging input.^15^, ^16^, ^17^, ^18^, ^19^ Unlike traditional saliency-based methods such as Gradient-weighted Class Activation Mapping, MM-LLMs can generate structured, clinically applicable outputs that, while not necessarily reflecting the internal reasoning of the model, present predictions in natural language and may improve the accessibility and usability of AI-based diagnostic assessments for clinicians.^20^, ^21^, ^22^, ^23^, ^24^ Despite their potential, these models often suffer from hallucinations, lack of quality-awareness, and rarely offer structured, sector-wise descriptions aligned with clinical OCT reports.^25^, ^26^, ^27^, ^28^

Existing approaches largely ignore the critical step of image quality assessment and do not emulate the structured format expected in ophthalmic documentation.^29^, ^30^, ^31^ Moreover, few models integrate multimodal data to produce clinically grounded, interpretable outputs.^32^^,^^33^ To address these limitations, we developed a fine-tuned MM-LLM capable of (1) automatically identifying unusable OCT scans, (2) detecting glaucoma from optic nerve head (ONH) circle scans, and (3) generating concise, structured clinical reports that include sector-wise RNFL thinning assessments.

Here, we fine-tuned an MM-LLM^34^^,^^35^ using a large dataset consisting of ONH OCT imaging paired with automatically generated clinical descriptions.^36^ The clinical descriptions incorporate text derived from OCT measurements of global and sectoral RNFL thinning, a glaucoma determination based on structural and functional measurements, and human review of OCT quality. The generated reports were used as ground truth during training and evaluation. By incorporating multiple sources of data, the models can be trained to provide clinical predictions beyond a simple summary of OCT results. Performance was evaluated across 3 tasks: image quality classification, glaucoma detection, and sector-wise RNFL thinning prediction. Clinical descriptions generated by the model were also evaluated using standard text evaluation metrics (BLEU, ROUGE, METEOR, and BERTScore). To our knowledge, this is the first MM-LLM designed specifically for structured ONH OCT report generation in glaucoma.

## Methods

### Data Description

This study draws upon imaging and clinical data collected through 2 well-established longitudinal cohorts: the Diagnostic Innovations in Glaucoma Study (ClinicalTrials.gov ID: NCT00221897)^37^ and the African Descent and Glaucoma Evaluation Study (ClinicalTrials.gov ID: NCT00221923).^38^ Both studies implemented harmonized, standard protocols and conducted serial ophthalmic evaluations, including OCT imaging and VF testing. The methodology and recruitment process were approved by the institutional review boards at each participating site, adhering to the ethical standards outlined in the Declaration of Helsinki. All participants provided written informed consent prior to enrollment.

A total of 43 849 Spectralis spectral-domain OCT (Heidelberg Engineering) circumpapillary B-scans, centered on the ONH, were included in this analysis. Scans were acquired with slightly varying diameters across different acquisition protocols over the study period (3.2–4.7 mm). The raw B-scan images, without segmentation overlays, were used as model input. The ground truth RNFL sector classifications were derived from the Spectralis device segmentation and normative database analysis. The University of California, San Diego Imaging Data Evaluation and Assessment Center assessed each scan to determine usability for clinical interpretation based on quality. This review consisted of experienced graders assessing scans for placement, centering, completeness, the presence of major artifacts, visibility of retinal layers, and substantial segmentation errors. As a result of the review, the graders assigned each scan a binary grade of usable or unusable.

Glaucomatous eyes were identified based on the presence of repeatable VF defects, defined as pattern standard deviation with *P* < 5% or Glaucoma Hemifield Test outside normal limits on at least 2 consecutive reliable examinations, or characteristic structural abnormalities of the ONH, such as neuroretinal rim thinning or localized RNFL loss, as determined by masked expert assessment of fundus photographs. Intraocular pressure was not used as a criterion for glaucoma labeling. Healthy eyes were required to have both normal VF results and normal optic disc appearance. Eyes showing discordant findings, such as normal fields with structural glaucomatous changes, were excluded to ensure diagnostic consistency.

Visual field testing was performed using the Humphrey Field Analyzer II, applying the 24-2 SITA Standard strategy. Tests exceeding established reliability thresholds, including fixation losses, false positive rates, or false negative rates greater than 33%, were excluded.

Glaucoma severity was classified based on visual field mean deviation (VF MD) from examinations within 1 year of the corresponding OCT scan: mild glaucoma was defined as VF MD better than –6 dB, and moderate-to-advanced glaucoma was defined as VF MD of –6 dB or worse.

### Structured Report Generation

**Figure 1:**
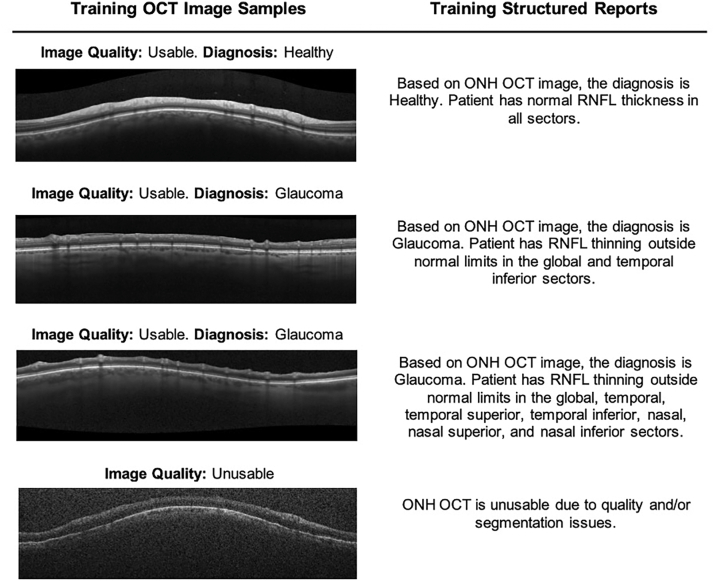
Samples of OCT circle scan images and corresponding automatically generated structured clinical reports were used for training of AI model. AI = artificial intelligence; ONH = optic nerve head; RNFL = retinal nerve fiber layer.

To facilitate supervised fine-tuning of the multimodal language model, structured clinical reports were automatically generated for each OCT circle scan based on corresponding diagnostic labels and sectoral RNFL classifications derived from the Spectralis report. It is important to note that the diagnostic labels (glaucoma vs. healthy) were determined independently from the RNFL sector classifications, as described above. These generated reports served as target text outputs during model training and were designed to emulate concise clinical documentation used in ophthalmic practice. Representative examples of these image-text training pairs are presented in Figure 1.

Each report incorporated 3 key components: the global glaucoma diagnosis (healthy or glaucoma), RNFL thinning status across 7 anatomical sectors (global, temporal, temporal superior, temporal inferior, nasal, nasal superior, and nasal inferior), and an image quality flag based on the standard University of California, San Diego Imaging Data Evaluation and Assessment Center assessment of the scans, indicating whether the scan was deemed usable for clinical interpretation.

Text templates were programmatically constructed to reflect the diagnostic interpretation of the scan. For example, if a scan was labeled as healthy and all RNFL sectors were within normal limits, the resulting report read: "Based on ONH OCT image, the diagnosis is Healthy. Patient has normal RNFL thickness in all sectors." In cases where sectoral thinning was observed, for instance, in the temporal and temporal superior regions, the description included specific mention of affected areas, such as: "Based on ONH OCT image, the diagnosis is Glaucoma. Patient has RNFL thinning outside normal limits in the temporal and temporal superior sectors." Sectoral RNFL classifications from the Spectralis normative database included within normal limits, borderline, and outside normal limits. To simplify the classification task, borderline classifications were grouped with within normal limits, resulting in a binary classification of each sector as within normal limits or outside normal limits.

For scans failing to meet the image quality criteria, a fixed refusal statement^39^ was assigned: "ONH OCT is unusable due to quality or segmentation issues." These standardized responses prevented the model from generating potentially misleading or speculative outputs when confronted with poor-quality input data.

The resulting paired dataset, composed of ONH OCT images and their automatically generated clinical descriptions, formed the foundation for supervised training.

### Model Architecture and Fine-Tuning Strategy

This study utilized the Llama 3.2 Vision-Instruct model, an 11-billion-parameter multimodal large language model (MM-LLM) capable of processing both text and image inputs. Llama 3.2 extends the architecture of the Llama 2 series by incorporating vision encoders and multimodal fusion modules, allowing for image-grounded language generation. The Vision-Instruct variant is instruction-tuned to follow text prompts while attending to visual features. Its architecture combines a transformer-based image encoder with a standard decoder-only large language model, connected via a multilayer feature projection and fusion network.^35^^,^^36^^,^^40^

We adopted the Unsloth implementation of the model, which supports parameter-efficient fine-tuning using LoRA (Low-Rank Adaptation)^41^ and QLoRA (Quantized LoRA).^42^ Fine-tuning was performed on the "unsloth/Llama-3.2-11B-Vision-Instruct-bnb-4bit" checkpoint using 4-bit quantized weights, allowing training to be conducted efficiently on limited GPU resources (one NVIDIA A40 GPU).^43^^,^^44^ During training, the vision encoder was kept frozen, while the language layers, attention modules, and multilayer perceptron layers were updated.

The model was fine-tuned on ONH OCT circle scans paired with structured clinical reports that were automatically generated based on Spectralis RNFL sector labels (within normal limits, borderline, and outside normal limits) and diagnostic ground truth. Each training example consisted of a single OCT image, a standard instruction prompt (“Describe the OCT scan in detail”), and the corresponding report as the output. A low temperature value (0.1) was used during inference to reduce randomness and encourage more deterministic, clinically consistent outputs. Hyperparameters used during training, including LoRA configuration and optimization settings, are detailed in Table S1, available at www.ophthalmologyscience.org. All model training and inference were performed offline on institutional computing infrastructure. No data were transmitted to external servers or third-party APIs.

### Model Evaluation

The fine-tuned Llama 3.2 Vision-Instruct model was evaluated on 3 key tasks: image quality triage, glaucoma detection, and sector-wise RNFL thinning classification. All assessments were conducted on a held-out test set (10% of all subjects) composed of ONH OCT scans excluded from model training.

For the image quality triage task, performance was measured by the model's ability to correctly identify scans that did not meet usability criteria and to generate an appropriate refusal statement. Glaucoma detection was evaluated by comparing the diagnostic impression in the generated report with the ground truth diagnosis based on standardized reading center criteria, as described earlier. Sector-wise RNFL thinning classification was assessed by matching results in the generated reports against corresponding Spectralis-derived labels in the global, temporal, temporal superior, temporal inferior, nasal, nasal superior, and nasal inferior sectors.

Classification performance across all tasks was quantified using standard metrics: accuracy, sensitivity, specificity, precision, and F1-score. A zero-rule baseline, representing the majority class, was used for comparison.

In addition to classification accuracy, the description quality of the generated structured reports was evaluated. As there is no consensus on which metric is best for evaluating AI-generated text to ground truth text, we used several different metrics, each focused on a particular aspect of text comparability and each with a range between 0 and 1 (high similarity).^45^, ^46^, ^47^, ^48^ These metrics have been widely adopted for clinical text evaluation, including applications in radiology and ophthalmology report generation, underscoring their relevance for medical and clinical domains in addition to general natural language processing.^49^, ^50^, ^51^, ^52^ BLEU (Bilingual Evaluation Understudy)^45^ quantifies the n-gram overlap between the generated and reference texts, with a focus on phrase-level precision. ROUGE (Recall-Oriented Understudy for Gisting Evaluation)^46^ emphasizes content recall and fluency, with ROUGE-1 and ROUGE-2 evaluating unigram and bigram matches, respectively, and ROUGE-L assessing sentence-level structural similarity via the longest common subsequence. METEOR^47^ accounts for synonymy and word order alignment, offering insights into semantic accuracy beyond exact lexical matches. BERTScore^48^ leverages contextual embeddings from pretrained language models to compute semantic similarity at a deeper, meaning-based level. Collectively, these metrics provide a robust, multidimensional assessment of how closely the model-generated reports align with expert-written clinical descriptions.

## Results

A total of 43 849 scans from 2198 eyes of 1310 subjects were divided into training/validation (1987 eyes from 1180 subjects; 40 103 scans) and testing (211 eyes from 130 subjects; 3746 scans) cohorts (Table 2). The mean number of scans per eye was 20.2 in the training/validation set and 17.8 in the testing set. The diagnostic distribution was comparable across splits, with glaucomatous eyes comprising 60.3% (1199 eyes) in the training/validation set and 62.6% (132 eyes) in the testing set. The mean (standard deviation) baseline age was 62.0 (15.0) years in the training/validation group and 59.9 (16.1) years in the testing group, with similar mean (standard deviation) last-visit ages (65.5 [0.9] vs. 63.1 [2.9] years [*P* value of 0.116], respectively). Most participants identified as White (51.5% and 47.7%) or Black/African American (40.8% and 46.2%), with smaller proportions identifying as Asian (∼5.3% in both training/testing sets), American Indian, or Pacific Islander. Sex distribution was relatively balanced, with females representing 59.0% of the training/validation and 56.9% of the testing cohort. Over 88% of participants in both groups identified as non-Hispanic. Mean ocular characteristics were also similar across cohorts, including axial length (∼24.2 mm), central corneal thickness (∼539–541 μm), intraocular pressure (∼14 mmHg), and VF MD (–5.14 vs. –5.50 dB), with no statistically significant differences observed. The distribution of RNFL thinning outside normal limits across sectors is also reported in Table 2. Thinning was most prevalent in the temporal inferior (43.3%), global (41.2%), and temporal superior (38.4%) sectors, while nasal sectors showed considerably lower rates (7.6%–13.3%).

**Table 2:** Comparison of Participant and Eye-Level Demographic and Ocular Characteristics between Cohort Splits

	Training and Validation (*n* = 1180 Subjects; 1987 Eyes; 40 103 Scans, 20.2 Mean Scans per Eye)	Testing (n = 130 Subjects; 211 Eyes; 3746 Scans, 17.8 Mean Scans per Eye)	*P* Value
Subject-level characteristics			
Baseline age, years	62.0 (61.1, 62.8)	59.9 (57.1, 62.7)	0.130
Last age, years	65.5 (64.6, 66.4)	63.1 (60.2, 66.0)	0.116
Race			
American Indian/Alaska Native	3 (0.3%)	0 (0.0%)	0.822
Asian	62 (5.3%)	7 (5.4%)	
Black or African American	482 (40.8%)	60 (46.2%)	
Native Hawaiian or Other Pacific Islander	3 (0.3%)	0 (0.0%)	
Unknown or not reported	22 (1.9%)	1 (0.8%)	
White	608 (51.5%)	62 (47.7%)	
Sex			
Female	696 (59.0%)	74 (56.9%)	0.707
Male	484 (41.0%)	56 (43.1%)	
Ethnicity			
Hispanic	37 (3.1%)	5 (3.8%)	0.606
Not Hispanic	1042 (88.3%)	117 (90.0%)	
Unknown or not reported	101 (8.6%)	8 (6.2%)	
Eye-level characteristics at latest imaging			
Axial length (mm)	24.2 (24.1, 24.2)	24.2 (24.0, 24.5)	0.715
CCT (μm)	538.6 (536.0, 541.2)	541.0 (533.3, 548.8)	0.558
24-2 VF MD (dB)	–5.14 (–5.52, –4.75)	–5.50 (–6.67, –4.34)	0.556
Spherical equivalent (D)	–0.67 (–0.81, –0.54)	–1.02 (–1.43, –0.61)	0.116
IOP (mmHg)	14.52 (14.26, 14.78)	14.02 (13.23, 14.81)	0.236
Diagnosis			
Glaucomatous	1199 (60.3%)	132 (62.6%)	0.956
Nonglaucomatous	788 (39.7%)	79 (37.4%)	
RNFL thinning outside normal limits (ONLs) distribution in different sectors, n (%)			
Global	16 249 (41.2%)	1516 (41.2%)	-
Temporal	6247 (15.9%)	700 (19.0%)	-
Temporal superior	15 134 (38.4%)	1642 (44.6%)	-
Temporal inferior	17 087 (43.3%)	1741 (47.3%)	-
Nasal	3729 (9.5%)	282 (7.7%)	-
Nasal superior	5266 (13.3%)	449 (12.2%)	-
Nasal inferior	3934 (10.0%)	281 (7.6%)	-

CCT = central corneal thickness; D = diopters; IOP = intraocular pressure; RNFL = retinal nerve fiber layer; VF MD = visual field mean deviation.

**Figure 2:**
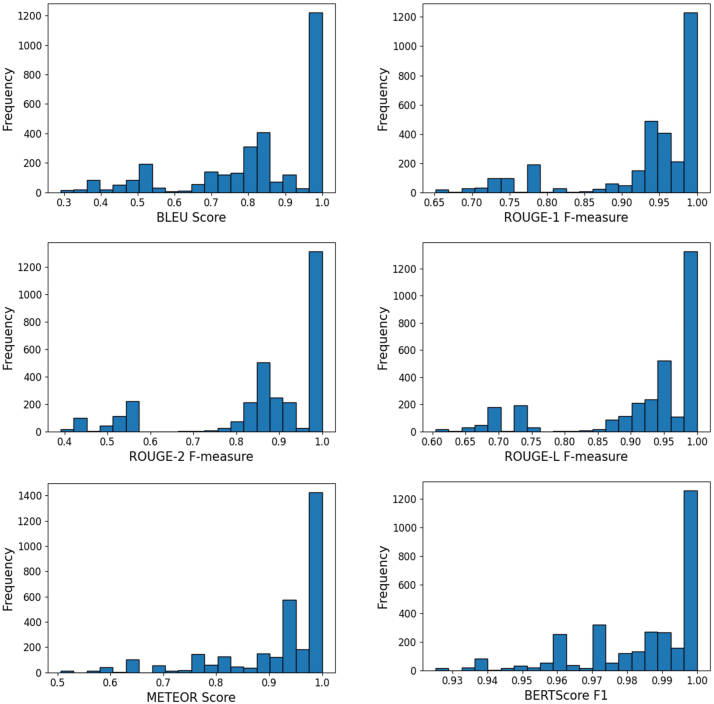
Distribution histograms of BLEU, ROUGE, METEOR, and BERTScore metrics for generated text descriptions.

The generated text descriptions demonstrated strong alignment with the reference reports across multiple evaluation metrics (Table 3, Fig 2). The model achieved an average BLEU score of 0.82 ± 0.19, reflecting high n-gram overlap. ROUGE-based evaluations further confirmed the quality of the outputs, with ROUGE-1, ROUGE-2, and ROUGE-L F-measures reaching 0.94 ± 0.08, 0.87 ± 0.17, and 0.92 ± 0.11, respectively, indicating consistency at the word, phrase, and sentence levels. METEOR scored 0.92 ± 0.11, suggesting effective handling of synonyms and word order. BERTScore_F1 was exceptionally high (0.99 ± 0.02), pointing to near-perfect semantic similarity between predicted and reference descriptions. Figure 2 demonstrates that the majority of generated descriptions closely align with the reference reports after excluding poor-quality images to focus solely on usable predictions.

**Table 3:** Summary of Text Description Evaluation Metrics (BLEU, ROUGE, METEOR, and BERTScore)

Metric	Value Mean (Standard Deviation)	Interpretation
BLEU Score	0.82 (0.19)	High n-gram overlap with the reference text, indicating strong word-level and phrase-level similarity.
ROUGE-1 F-measure	0.94 (0.08)	Excellent unigram recall, showing that most individual words match the reference text (word-level).
ROUGE-2 F-measure	0.87 (0.17)	Strong bigram overlap, reflecting the model’s ability to capture phrase-level coherence.
ROUGE-L F-measure	0.92 (0.11)	High similarity in the longest common subsequence, suggesting well-preserved sentence-level structure.
METEOR	0.92 (0.11)	Incorporates synonymy and word order alignment, indicating semantically accurate and fluent descriptions.
BERTScore_F1	0.99 (0.02)	Extremely high semantic similarity based on contextual embeddings, showing alignment in meaning beyond surface-level text.

**Figure 3:**
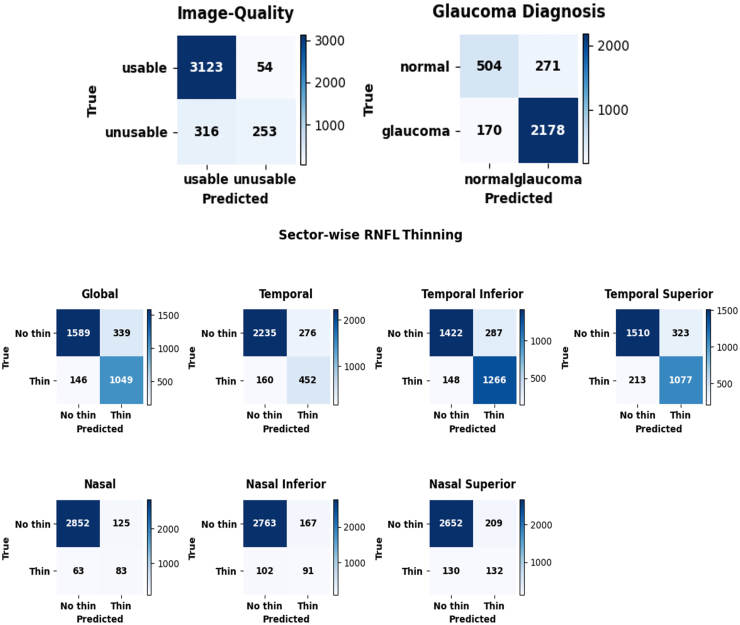
Confusion matrices for image quality detection, glaucoma detection, and 7 sector-wise RNFL thinning predictions. RNFL = retinal nerve fiber layer.

The model demonstrated strong performance across all classification tasks (Table 4), with class-level outcomes visualized in the confusion matrices (Fig 3). For image quality assessment, it achieved 0.90 accuracy, surpassing the zero-rule baseline of 0.85, with high specificity (0.98) but low sensitivity (0.44). In glaucoma diagnosis, the model reached an accuracy of 0.86 and an F1-score of 0.91, outperforming the zero-rule baseline (0.75). For sector-wise RNFL thinning prediction, accuracies ranged from 0.83 to 0.94. The model particularly excelled in the global and temporal sectors, especially the temporal superior and inferior, where it significantly exceeded the zero-rule baselines. For example, model performance was higher compared to the zero-rule baseline in the global (0.84 vs. 0.62), temporal inferior (0.86 vs. 0.55), and temporal superior (0.83 vs. 0.59) sectors. These results highlight the model’s effectiveness in detecting RNFL thinning in commonly affected regions. In contrast, while the model showed high accuracy in the nasal sectors (0.89–0.94), these values were close to or slightly below the zero-rule baselines.

**Table 4:** Model Performance Evaluation across Image Quality, Glaucoma Diagnosis, and Sector-Wise RNFL Thinning Prediction

Feature	Accuracy	Sensitivity	Specificity	Precision	F1-Score	Zero-Rule Baseline∗
Image quality	0.90 (0.87, 0.93)	0.44 (0.32, 0.58)	0.98 (0.97, 0.99)	0.82 (0.73, 0.90)	0.58 (0.45, 0.690)	0.85
Glaucoma diagnosis	0.86 (0.81, 0.90)	0.93 (0.88, 0.96)	0.65 (0.55, 0.76)	0.89 (0.82, 0.94)	0.91 (0.86, 0.94)	0.75
Sector-wise RNFL thinning prediction:						
Global	0.84 (0.80, 0.89)	0.88 (0.79, 0.94)	0.82 (0.75, 0.90)	0.76 (0.66, 0.85)	0.81 (0.74, 0.88)	0.62
Temporal	0.86 (0.81, 0.90)	0.74 (0.59, 0.86)	0.89 (0.85, 0.93)	0.62 (0.42, 0.77)	0.67 (0.52, 0.79)	0.80
Temporal inferior	0.86 (0.81, 0.91)	0.90 (0.83, 0.95)	0.83 (0.76, 0.90)	0.82 (0.73, 0.89)	0.85 (0.79, 0.91)	0.55
Temporal superior	0.83 (0.79, 0.87)	0.83 (0.77, 0.89)	0.824 (0.77, 0.8)	0.77 (0.69, 0.84)	0.80 (0.74, 0.85)	0.59
Nasal	0.94 (0.91, 0.96)	0.57 (0.26, 0.80)	0.96 (0.94, 0.98)	0.40 (0.19, 0.56)	0.47 (0.24, 0.62)	0.95
Nasal inferior	0.91 (0.88, 0.95)	0.47 (0.24, 0.71)	0.94 (0.92, 0.97)	0.35 (0.14, 0.59)	0.40 (0.19, 0.61)	0.94
Nasal superior	0.89 (0.85, 0.93)	0.50 (0.32, 0.65)	0.93 (0.89, 0.96)	0.39 (0.21, 0.55)	0.44 (0.27, 0.56)	0.92

RNFL = retinal nerve fiber layer.

∗ The zero-rule baseline: Predicts the majority class. Serves as a baseline for model performance, particularly in imbalanced datasets.

**Figure 4:**
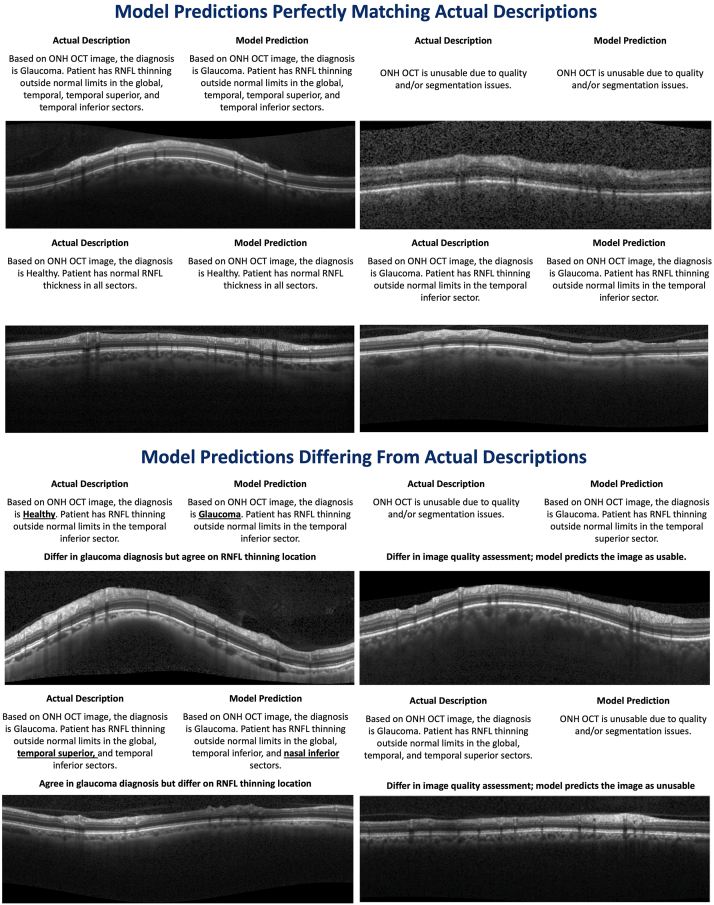
Examples of acceptable and unacceptable or unusable quality scans (with a refusal statement) with corresponding actual and model reports. ONH = optic nerve head; RNFL = retinal nerve fiber layer.

Figure 4 also presents qualitative examples of accepted and refused scans, as well as cases where the model’s predictions either closely matched or diverged from the actual clinical descriptions.

In the stratified analysis by glaucoma severity (Table 5), diagnostic and image quality classification performance remained consistent across severity groups, with image quality accuracy at 0.90 for mild and 0.86 for moderate-to-advanced glaucoma, and glaucoma diagnosis accuracy at 0.81 in both groups. However, the model’s performance in RNFL thinning prediction varied notably across circumpapillary sectors. In moderate-to-advanced glaucoma, accuracy reached 0.94 in the global sector, 0.87 in the temporal superior, and 0.97 in the temporal inferior sector, substantially higher than corresponding accuracies in mild cases (0.76, 0.77, and 0.80, respectively) with *P* value < 0.001. Conversely, in the nasal sectors, the model performed significantly better in mild glaucoma, with accuracies of 0.92 (nasal superior), and 0.94 (nasal inferior), compared to 0.71, and 0.78 in the moderate-to-advanced group (*P* value < 0.001). These findings suggest the model excels at detecting pronounced thinning in advanced glaucoma, while improvements are needed to enhance sensitivity to early-stage changes, particularly in less affected nasal regions. Based on Table S6, available at www.ophthalmologyscience.org, no significant differences in diagnostic performance were observed across different age groups.

**Table 5:** Stratified Diagnostic Accuracy (95% CI) of the MM-LLM across Glaucoma Severity Groups (Mild vs. Moderate-to-Advanced) for Classification Tasks Using OCT Circle Scans

Glaucoma Severity	Mild Glaucoma (*n* = 62 Subjects, 81 Eyes, 1781 Scans, 22.0 Mean Scans per Eye)			Moderate-to-Advanced Glaucoma (*n* = 40 Subjects, 56 Eyes, 891 Scans, 15.9 Mean Scans per Eye)		
Feature	Accuracy	Sensitivity	Specificity	Accuracy	Sensitivity	Specificity
Image quality	0.90 (0.84, 0.92)	0.30 (0.19, 0.37)	0.99 (0.97, 0.99)	0.86 (0.84, 0.86)	0.51 (0.35, 0.68)	0.97 (0.95, 0.98)
Glaucoma diagnosis	0.81 (0.74, 0.87)	0.89 (0.83, 0.94)	0.65 (0.55, 0.76)	0.81 (0.72, 0.88)	0.99 (0.97, 1.00)	0.65 (0.55, 0.75)
RNFL thinning:						
Global	0.76 (0.68, 0.84)	0.80 (0.66, 0.91)	0.74 (0.63, 0.85)	0.94 (0.84, 0.97)	0.97 (0.92, 0.99)	0.56 (0.22, 0.84)
Temporal	0.85 (0.79, 0.90)	0.57 (0.40, 0.78)	0.88 (0.82, 0.93)	0.80 (0.69, 0.87)	0.89 (0.78, 0.95)	0.69 (0.55, 0.79)
Temporal superior	0.77 (0.73, 0.81)	0.76 (0.65, 0.86)	0.77 (0.70, 0.84)	0.87 (0.79, 0.93)	0.96 (0.91, 0.99)	0.41 (0.18, 0.65)
Temporal inferior	0.80 (0.71, 0.88)	0.86 (0.77, 0.93)	0.76 (0.62, 0.87)	0.97 (0.93, 0.99)	0.99 (0.99, 1.00)	0.71 (0.43, 0.90)
Nasal	0.96 (0.94, 0.98)	0.19 (0.00, 0.36)	0.97 (0.95, 0.99)	0.87 (0.81, 0.93)	0.86 (0.70, 0.94)	0.87 (0.79, 0.94)
Nasal superior	0.92 (0.87, 0.96)	0.16 (0.000, 0.4)	0.96 (0.93, 0.99)	0.71 (0.63, 0.78)	0.70 (0.60, 0.82)	0.72 (0.61, 0.80)
Nasal inferior	0.94 (0.88, 0.97)	0.22 (0.00, 0.65)	0.97 (0.94, 0.99)	0.78 (0.69, 0.84)	0.63 (0.33, 0.83)	0.80 (0.72, 0.88)

CI = confidence interval; MM-LLM = multimodal large language model; RNFL = retinal nerve fiber layer; VF MD = visual field mean deviation.

Mild glaucoma: VF MD > –6 dB; moderate-to-advanced glaucoma: VF MD ≤ –6 dB. Severity was classified based on the paired VF examination within 1 year of the OCT scan.

Figures S5 and S6, available at www.ophthalmologyscience.org present a comparison between the fine-tuned model and the original, nonfine-tuned Llama 3.2 model. When prompted with a general instruction, the original model often generates vague and nonspecific descriptions, lacking the diagnostic precision required in clinical settings. Even when guided by the structured prompt (mirroring the format used during fine-tuning) and evaluated with a low temperature setting (0.1), the original model frequently defaults to labeling all images as “healthy,” RNFL thickness as “within normal limits,” and image quality as “usable.” These findings underscore the importance of domain-specific fine-tuning in enabling the model to generate accurate, structured, and clinically meaningful ONH OCT reports.

## Discussion

This study demonstrates that fine-tuned MM-LLMs can generate structured clinical reports from OCT scans with high accuracy across multiple evaluation metrics. Importantly, the objective of this study was not to replace the structured outputs already available from the Spectralis device. The automatically generated reports incorporated not only OCT results (e.g., global and sectoral RNFL thinning) but also glaucoma status and human-graded quality. These additional sources of information allowed the models to be trained to go beyond just providing an automated summary of OCT results. They also provide predictions of overall glaucoma status and of quality issues often not captured by quantitative OCT quality metrics, such as artifacts, imaging misalignments, and segmentation errors. By delivering both accurate glaucoma detection and clinically grounded interpretability, these models represent a significant step toward the integration of AI-assisted diagnostics into clinical practice.

By generating structured, human-like clinical reports from OCT scans, the model not only achieves high diagnostic accuracy but also provides explanations that align closely with clinical reasoning. This reasoning-based interpretability helps bridge the gap between AI predictions and clinician judgment, potentially improving diagnostic confidence and patient care. The report generated by the model could also serve as a draft for clinician documentation, with potential to make clinical workflows more efficient and reduce documentation burden for ophthalmologists.

The model also provides only a binary quality determination (usable vs. unusable). While this is useful to help quickly determine whether a patient’s OCT will be useful for glaucoma assessment, or re-imaging may be necessary, the lack of a cause-specific quality determination may limit clinical usefulness in some ways (e.g., when another pathology is causing quality issues). Poor-quality OCT scans can mislead AI models and trigger hallucinated outputs, statements that sound plausible but are clinically inaccurate.^53^, ^54^, ^55^ By automatically identifying unusable scans and returning a fixed refusal statement, the model avoids producing speculative interpretations based on unreliable inputs. This safeguard is critical for clinical deployment, where erroneous outputs may misguide decision-making or erode trust in AI systems.^31^ Quality triage ensures that generated reports are grounded in diagnostically valid data, helping to prevent misleading interpretations from poor-quality scans and thus supporting model transparency.

The structured text outputs generated by the model offer a format that is more clinically applicable than traditional visualization methods such as Gradient-weighted Class Activation Mapping, which produce coarse heatmaps without explicit diagnostic context.^56^^,^^57^ In contrast, MM-LLMs provide predictions (and supporting output) in natural language, generating more actionable output for clinicians.^22^^,^^23^

We opted to freeze the vision encoder during training to reduce computational overhead and prevent the loss of general visual representations. This design choice, supported by prior multimodal architectures,^58^, ^59^, ^60^, ^61^ allowed the language components to adapt effectively to clinical report generation while preserving robust image embeddings.

The model consistently performed well across evaluation metrics, demonstrating its ability to generate accurate and semantically rich reports from ONH OCT scans. High BLEU and ROUGE scores suggest strong syntactic alignment with reference reports, while elevated METEOR and BERTScore values highlight the model’s grasp of semantic content. Part of this strong performance likely stems from the structured nature of the target reports, enabling the model to learn consistent templates and improve similarity metrics.

Performance was especially strong in the temporal superior, temporal inferior, and global sectors. In contrast, the model performance was worse (i.e., no better than the zero-rule baseline) in the nasal sectors. This may be the result of class imbalance within the datasets used here. As shown in Table 2, the prevalence of RNFL thinning outside normal limits in nasal sectors ranged from 7.6% to 13.3%, compared to 38.4% to 47.3% in temporal superior, temporal inferior, and global sectors. These findings highlight the impact of differences in class balance across different anatomical regions within a single dataset.

The comparison between the fine-tuned and original models highlights the essential role of fine-tuning in producing accurate and clinically meaningful outputs. As shown in the supplementary figures, the original model often generates vague descriptions and misclassifies low-quality scans as usable. Fine-tuning significantly improves both diagnostic precision and image quality assessment, ensuring outputs align with structured clinical standards. These findings emphasize the necessity of domain-specific fine-tuning for reliable medical applications of large language models.^62^^,^^63^

Beyond the challenges associated with OCT-based interpretation, additional limitations merit consideration. This study relied solely on a single circumpapillary B-scan, which does not capture the full range of structural parameters used in comprehensive glaucoma evaluation, such as volumetric ONH analysis, thickness and deviation maps, or macular ganglion cell layer and inner plexiform layer assessments. Although the circumpapillary RNFL scan remains one of the most widely used measures in glaucoma assessment, the scope of this model is limited to this single modality and should be interpreted as a foundational step toward more comprehensive automated OCT report generation.

The current model also generates qualitative (not quantitative) categorical descriptions of RNFL status at a single time point, which may limit applicability for longitudinal follow-up and progression monitoring. Additionally, the quality assessment module achieved low sensitivity (0.44), although with high specificity (0.98), indicating that a substantial proportion of poor-quality scans were not detected. The module also provides a binary classification of scan usability without specifying the underlying cause, such as low signal strength, segmentation failure, or comorbidities like vitreomacular traction. Improving both detection sensitivity and providing more granular quality feedback remain important considerations. Similarly, the specificity for glaucoma detection (0.73) indicates that a notable proportion of healthy eyes were misclassified as glaucomatous, which would be a particular concern in screening settings where disease prevalence is low and false positives may lead to unnecessary referrals.

Notably, in some cases, the model's predictions of RNFL thinning appeared more consistent with the clinical presentation than the device-derived ground truth labels, suggesting that the MM-LLM may capture patterns not fully reflected in automated segmentation. Formal adjudication of such discordant cases may help refine ground truth labels in future studies.

Training on structured clinical reports may cause the model to overfit templated phrasing, potentially reducing adaptability to varied clinical documentation styles. However, these reports may potentially offer more detail and usable information than existing clinician-generated reports, which are often brief due to the high volume of patient encounters typical in ophthalmic practice.

The dataset is enriched for glaucomatous eyes due to the nature of the Diagnostic Innovations in Glaucoma Study and African Descent and Glaucoma Evaluation Study cohorts, and this distribution does not reflect the prevalence of glaucoma in general clinical or screening populations, where healthy and suspect eyes substantially outnumber confirmed glaucoma cases. Model performance may therefore differ in clinical settings with lower disease prevalence. Moreover, observed performance disparities across circumpapillary sectors highlight the model's sensitivity to imbalanced training data, as the prevalence of RNFL thinning in nasal sectors was substantially lower than in temporal and global sectors (Table 2).

The study population also did not include highly myopic eyes, as the Diagnostic Innovations in Glaucoma Study/African Descent and Glaucoma Evaluation Study originally excluded highly myopic eyes. Since myopia is known to be independently associated with alterations in ONH morphology, the model's performance in these eyes has not yet been evaluated. Future work will incorporate additional highly myopic eyes in training and testing datasets.

An important limitation is the exclusion of glaucoma suspects and borderline cases, which was done to ensure unambiguous diagnostic labels for training. However, in general clinical populations, such cases are commonly encountered and represent a critical diagnostic challenge. The current model is limited in its ability to handle borderline diagnostic categories.

Although the model does not receive patient demographic information such as age, the Spectralis normative database used to derive the ground truth labels is age-dependent, meaning that age-related information is implicitly encoded in the training labels. Whether the model captures age-related RNFL appearance patterns from the images or reflects the age distribution of the training data cannot be determined from the current analysis. No statistically significant differences in performance were observed across age groups (Table S6, available at www.ophthalmologyscience.org), though the wide confidence intervals suggest the test set may be underpowered to detect such effects.

To address these concerns, future research should incorporate diverse and balanced datasets, explore cross-institutional transfer learning, and include racially and ethnically representative populations. Future work should also incorporate suspect and preperimetric glaucoma cases, quantitative thickness predictions, and longitudinal modeling capabilities. Integrating complementary modalities, such as fundus photographs, VF tests, volumetric OCT data, and macular structural assessments, may further enhance diagnostic accuracy and support more comprehensive clinical evaluation.

As MM-LLMs advance toward clinical adoption, ensuring transparency, fairness, and human oversight is critical. Embedding interpretable reasoning in AI outputs is not only a technical strength but also a clinical necessity to mitigate automation bias and uphold patient–clinician trust.

## Conclusion

This study demonstrates the potential of fine-tuned multimodal language models to generate structured, interpretable clinical reports from OCT scans with high diagnostic accuracy. By integrating a quality triage mechanism, the model reduces misleading outputs from poor-quality scans, supporting safety and transparency. The model's sentence-level outputs explicitly localize RNFL thinning across anatomical sectors, improving the readability and clinical usability of AI-generated OCT reports. These features position our approach as a scalable solution for glaucoma decision support, as well as a potential approach for reducing clinical documentation burden. Future work integrating diverse datasets and multimodal inputs will further enhance generalizability and support safe clinical deployment.
