# Supplementary material for: Image-Quality–Aware Multimodal Artificial Intelligence for Automated Structured OCT Report Generation in Glaucoma Evaluation
Source: Ophthalmol Sci. 2026 May 26;6(8):101254. doi: 10.1016/j.xops.2026.101254 (PMC13343140; doi:10.1016/j.xops.2026.101254)
Supplement: Figure S5 [file mmc1.pdf]

A

General and structured prompt setups for generating outputs with the non-fine-tuned Llama 3.2 model on OCT circle scan images

🔹 **General Prompt Setup**

System Prompt:
You are a knowledgeable assistant for medical imaging.

User Prompt:
Describe the OCT scan in detail!

🔸 **Structured Prompt Setup**

System Prompt:
You are a clinical assistant trained to generate structured ONH OCT reports. Each report must follow this format:

Diagnosis: [Healthy or Glaucoma]
RNFL Assessment:

Global: [within or outside of normal limits]
Temporal: [within or outside of normal limits]
Temporal Superior: [within or outside of normal limits]
Temporal Inferior: [within or outside of normal limits]
Nasal: [within or outside of normal limits]
Nasal Superior: [within or outside of normal limits]
Nasal Inferior: [within or outside of normal limits]
Image Quality: [Usable / Unusable due to quality issues]

Generate your response based solely on the image and do not speculate beyond what is visible.

User Prompt:
Describe the OCT scan in detail!

B

Comparison between the outputs of fine-tuned and non-fine-tuned Llama 3.2 models

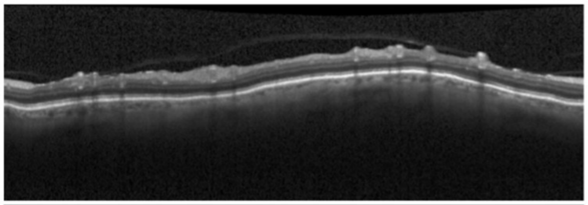

Actual Description: Based on ONH OCT image, the diagnosis is Glaucoma. Patient has RNFL thinning outside normal limits in the global, temporal superior, and temporal inferior sectors. **Fine-tuned Llama:**Based on ONH OCT image, the diagnosis is Glaucoma. Patient has RNFL thinning outside normal limits in the global, temporal superior, and temporal inferior sectors. **Non-fine-tuned Llama with General Prompt:**An OCT (Optical Coherence Tomography) scan is a non-invasive imaging test that uses low-coherence interferometry to capture high-resolution images of the retina and other structures in the eye. It is commonly used to diagnose and monitor various eye conditions, such as age-related macular degeneration, diabetic retinopathy, and glaucoma. **What is shown in the OCT scan? ** The OCT scan shows a cross-sectional image of the retina, which is the light-sensitive tissue at the back of the eye. The image is composed of multiple layers, each representing different structures within the retina. **Layers of the retina: **Retinal pigment epithelium (RPE): ** The outermost layer of the retina, responsible for absorbing excess light and maintaining the health of the photoreceptors. * **Choroid: ** A layer of blood vessels that supplies oxygen and nutrients to the retina. ***Sclera: ** The white, fibrous tissue that surrounds the eye and provides protection. * **Vitreous humor: ** The clear gel-like substance that fills the space between the lens and the retina. **Abnormalities in the OCT scan: **Retinal thinning: ** The retina appears thinner than normal, which can be a sign of age-related macular degeneration or other retinal diseases. * **Fluid accumulation: ** Fluid accumulation in the retina, which can be a sign of diabetic retinopathy or other conditions. * **Scarring: ** Scarring or atrophy of the retina, which can be a sign of previous eye injury or disease. **Interpretation of the OCT scan: ** The OCT scan provides valuable information about the structure and health of the retina. It can help diagnose various eye conditions and monitor their progression over time. The scan can also guide treatment decisions and monitor the effectiveness of therapy. **Conclusion: ** The OCT scan is a powerful diagnostic tool that provides detailed images of the retina and other structures in the eye. It can help diagnose and monitor various eye conditions and guide treatment decisions. **Non-fine-tuned Llama with Structured Prompt Similar to Actual Reports:**Diagnosis: Healthy **RNFL Assessment: ** - Global: Within normal limits - Temporal: Within normal limits - Temporal Superior: Within normal limits - Temporal Inferior: Within normal limits - Nasal: Within normal limits - Nasal Superior: Within normal limits – Nasal Inferior: Within normal limits **Image Quality:

** Usable

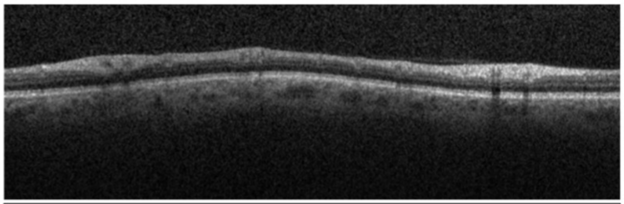

Actual Description: ONH OCT is unusable due to quality and/or segmentation issues.

Fine-tuned Llama: ONH OCT is unusable due to quality and/or segmentation issues.

Non-fine-tuned Llama with General Prompt: The provided OCT (Optical Coherence Tomography) scan is a cross-sectional image of the retina, showcasing the layered structure of the eye's inner surface. The scan displays a horizontal section through the retina, with the top of the image representing the surface of the retina and the bottom representing the choroid, a layer of blood vessels between the sclera and retina. The choroid is a thin, vascular layer that supplies oxygen and nutrients to the retina. The scan reveals a normal retinal structure, with distinct layers visible, including the retinal pigment epithelium (RPE), photoreceptor layer, outer nuclear layer, outer plexiform layer, inner nuclear layer, inner plexiform layer, ganglion cell layer, and nerve fiber layer. These layers are essential for transmitting visual information from the retina to the brain. The scan also shows the presence of a few small drusen, which are small yellow deposits that can accumulate under the retina and are associated with age-related macular degeneration (AMD). However, the drusen are not large enough to cause significant vision loss at this stage. Overall, the OCT scan provides valuable information about the health of the retina and can help diagnose and monitor various retinal conditions, such as AMD, diabetic retinopathy, and macular edema.

Non-fine-tuned Llama with Structured Prompt Similar to Actual Reports:
Diagnosis: Healthy **RNFL Assessment: ** - Global: Within normal limits - Temporal: Within normal limits - Temporal Superior: Within normal limits - Temporal Inferior: Within normal limits - Nasal: Within normal limits - Nasal Superior: Within normal limits – Nasal

Supplementary Figure S5: A: Prompt setups for non-fine-tuned Llama 3.2 on OCT circle scans. B: Comparison of fine-tuned vs. non-fine-tuned Llama 3.2 outputs.
