# Supplementary material for: Image-Quality–Aware Multimodal Artificial Intelligence for Automated Structured OCT Report Generation in Glaucoma Evaluation
Source: Ophthalmol Sci. 2026 May 26;6(8):101254. doi: 10.1016/j.xops.2026.101254 (PMC13343140; doi:10.1016/j.xops.2026.101254)
Supplement: Figure S6 [file mmc2.pdf]

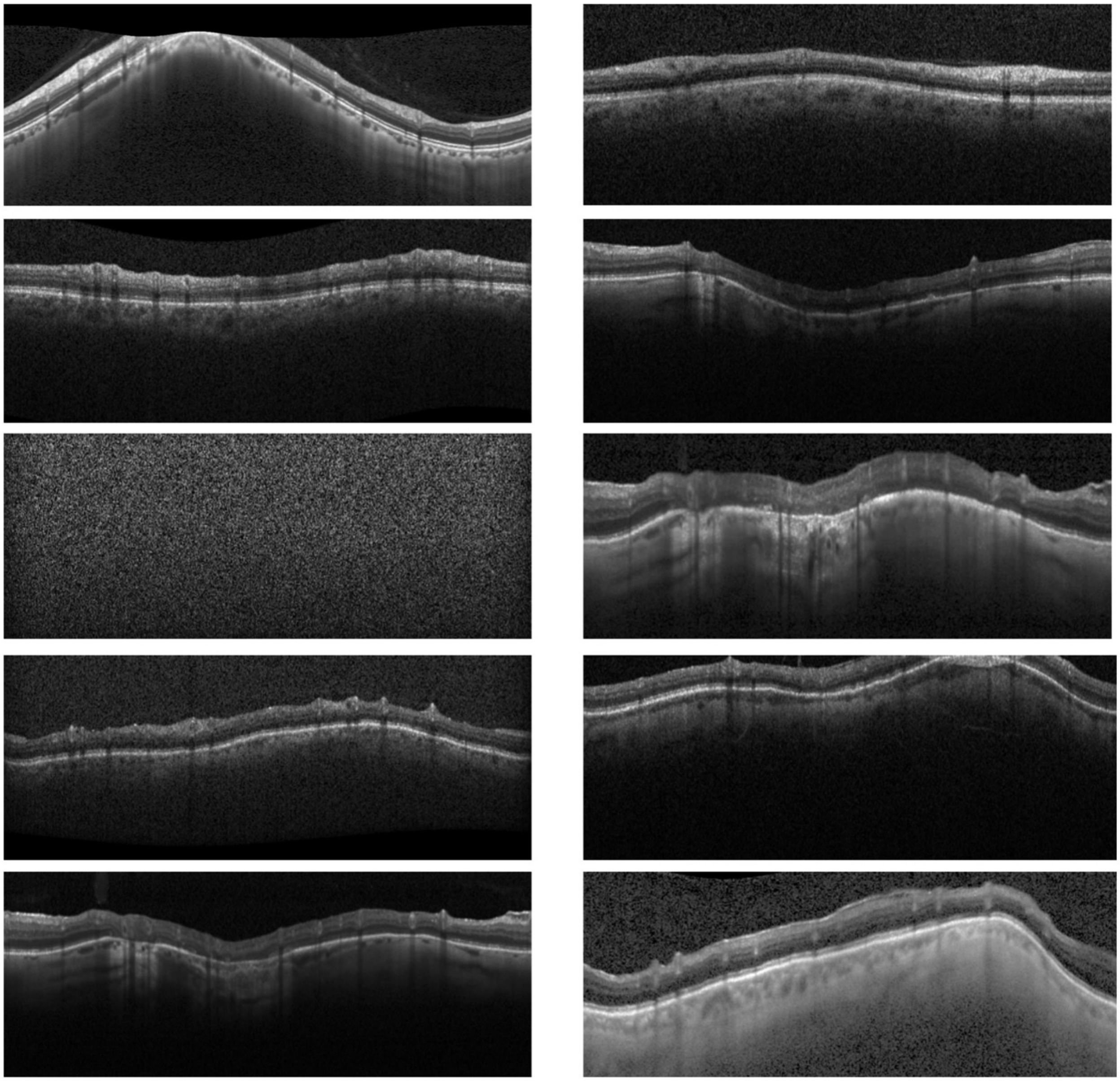

Supplementary Figure S6: Examples of poor quality scans where both ground truth and the fine-tuned model correctly identified ONH OCT scans as unusable, while the original Llama 3.2 model incorrectly labeled them as usable, underscoring the need for domain-specific instruction tuning.
