# Supplementary material for: Image-Quality–Aware Multimodal Artificial Intelligence for Automated Structured OCT Report Generation in Glaucoma Evaluation
Source: Ophthalmol Sci. 2026 May 26;6(8):101254. doi: 10.1016/j.xops.2026.101254 (PMC13343140; doi:10.1016/j.xops.2026.101254)
Supplement: Table S1 [file mmc3.pdf]

Supplementary Table S1: Fine-tuning hyperparameters	
**Parameter**	Value
**Base model**	unsloth/Llama-3.2-11B-Vision-Instruct-bnb-4bit
**Precision**	4-bit (QLoRA)
**LoRA rank (r)**	16
**LoRA alpha**	16
**LoRA dropout**	0.0
**Finetuned modules**	Language layers, MLP, attention
**Frozen modules**	Vision encoder
**Batch size**	2 per device
**Gradient accumulation steps**	4
**Number of epochs**	3
**Learning rate**	2e-4
**Optimizer**	AdamW (8-bit)
**Weight decay**	0.01
**Scheduler**	Linear
**Max sequence length**	2048
**Instruction prompt**	"Describe the OCT scan in detail."
