# Supplementary material for: Image-Quality–Aware Multimodal Artificial Intelligence for Automated Structured OCT Report Generation in Glaucoma Evaluation
Source: Ophthalmol Sci. 2026 May 26;6(8):101254. doi: 10.1016/j.xops.2026.101254 (PMC13343140; doi:10.1016/j.xops.2026.101254)
Supplement: Table S6 [file mmc4.pdf]

Supplementary Table S6: Stratified diagnostic accuracy (95% CI) of the MM-LLM across different age groups for classification tasks using OCT circle scans

**Age**	**Age > Median (65.3 years) (n = 65 subjects)**			**Age < Median (65.3 years) (n = 79 subjects)**		
**Feature**	Accuracy	Sensitivity	Specificity	Accuracy	Sensitivity	Specificity
**Image Quality**	0.92 (0.88, 0.95)	0.60 (0.42, 0.75)	0.98 (0.96, 0.99)	0.88 (0.84, 0.92)	0.28 (0.18, 0.41)	0.99 (0.98, 1.00)
**Glaucoma Diagnosis**	0.92 (0.86, 0.96)	0.96 (0.92, 0.99)	0.52 (0.38, 0.66)	0.80 (0.72, 0.87)	0.88 (0.80, 0.94)	0.68 (0.55, 0.81)
**RNFL Thinning:**						
**Global**	0.78 (0.71, 0.86)	0.86 (0.75, 0.95)	0.70 (0.59, 0.83)	0.91 (0.85, 0.95)	0.91 (0.79, 0.96)	0.91 (0.84, 0.96)
**Temporal**	0.79 (0.72, 0.86)	0.66 (0.46, 0.86)	0.82 (0.75, 0.89)	0.93 (0.89, 0.96)	0.82 (0.62, 0.92)	0.96 (0.93, 0.98)
**Temporal Superior**	0.78 (0.73, 0.82)	0.85 (0.76, 0.92)	0.71 (0.63, 0.79)	0.88 (0.83, 0.92)	0.82 (0.69, 0.92)	0.91 (0.86, 0.95)
**Temporal Inferior**	0.82 (0.74, 0.90)	0.90 (0.81, 0.97)	0.69 (0.58, 0.83)	0.90 (0.85, 0.95)	0.89 (0.77, 0.97)	0.91 (0.86, 0.95)
**Nasal**	0.94 (0.91, 0.96)	0.39 (0.05, 0.64)	0.96 (0.94, 0.98)	0.94 (0.89, 0.98)	0.67 (0.16, 0.96)	0.96 (0.91, 0.99)
**Nasal Superior**	0.87 (0.82, 0.92)	0.40 (0.13, 0.75)	0.90 (0.84, 0.95)	0.91 (0.86, 0.95)	0.56 (0.38, 0.69)	0.96 (0.91, 0.98)
**Nasal Inferior**	0.90 (0.84, 0.94)	0.32 (0.18, 0.68)	0.93 (0.89, 0.96)	0.93 (0.87, 0.97)	0.60 (0.17, 0.82)	0.95 (0.91, 0.99)
